# Transforaminal lumbar interbody fusion with an expandable interbody device: Two-year clinical and radiographic outcomes

**DOI:** 10.1016/j.xnsj.2023.100286

**Published:** 2023-10-13

**Authors:** Marc A. Weinstein, Giovanni A. Ayala, Raúl Roura, Kaitlyn N. Christmas, Deborah H. Warren, Peter Simon

**Affiliations:** a Department of Orthopaedics and Sports Medicine of the University of South Florida Morsani School of Medicine, 13330 USF Laurel Drive, Tampa, FL, USA; b Florida Orthopaedic Institute, 13020 Telecom Prkw. N., Tampa, FL, USA; c Foundation for Orthopaedic Research and Education, 4115 W Spruce St, Tampa, FL 33607, USA

**Keywords:** Expandable cage, TLIF, Lumbar interbody fusion, Radiographic parameters, Back pain, Lumbar lordosis, Segmental lordosis

## Abstract

**Background:**

The use of interbody cages as an adjunct to lumbar spinal fusion remains an important technique to enhance segmental stability, promote solid arthrodesis, maintain neuroforaminal decompression, and preserve/improve segmental lordosis. Appropriate segmental lumbar lordosis and sagittal balance is well-known to be critical for long-term patient outcomes. This study sought to evaluate the radiographic and clinical results of TLIF in patients using an articulating, expandable cage. Primary endpoint was clinical and radiographic outcomes, including complications, at 12 and 24 months.

**Methods:**

A total of 37 patients underwent open single-level or 2-level TLIF by a single surgeon using an expandable cage with concomitant bilateral pedicle screws and posterolateral arthrodesis. Clinical outcomes included ODI and VAS for back and legs. Radiographic outcomes included pelvic incidence and tilt, lumbar and segmental lordoses, and disc height at the operative level(s). All outcomes were collected at baseline, 2-weeks, 6-weeks, 3-months, 6-months, 12-months, and 24-months postop.

**Results:**

A total of 28 patients were available for analysis. Nine patients failed to follow-up at 24 months. Mean ODI scores showed significant improvement, from pre-to-postoperative at 24 months (55%; p<.0001). VAS for back and legs was significantly lower at 24 months on average by 72 and 79%, respectively (p<.0001 for both). Both segmental and lumbar lordoses significantly improved by 5.3° and 4.2° (p<.0001 and p=.049), respectively. Average disc height improved by 49% or 6.1 mm (p<.001). No device-related complications nor instances of measured subsidence. One patient had a superficial infection, and another had an intraoperatively repaired incidental durotomy.

**Conclusions:**

The use of an expandable cage contributed to improvement in both segmental and lumbar lordosis with no reported complications at 24-month follow-up. All clinical measures significantly improved as well. The expandable cage design represents an effective and safe option to increase cage size and allow significant segmental lordosis correction.

## Background

The use of interbody cages as an adjunct to lumbar spinal fusion remains an important technique to enhance segmental stability, promote solid arthrodesis, maintain neuroforaminal decompression, and improve/preserve segmental lordosis [1], [2], [3], [4], [5]. Appropriate segmental lumbar lordosis and sagittal balance is well-known to be critical for long-term patient outcomes after arthrodesis [6,7]. In addition, it is important to maintain and, if necessary, improve lumbar lordosis even in short-segment degenerative fusions [8], [9], [10], [11].

The transforaminal interbody approach to lumbar decompression and fusion (TLIF) allows for direct visualization and decompression of the neural elements during a single procedure [12,13]. However, the placement of a large anteriorly directed interbody device from a posterior approach has inherent difficulties due to anatomic considerations required to create or maintain lordosis [14], [15], [16]. There is risk of damage to the neural elements during insertion, destruction of the end plates from cage impaction, potential for collapse of the disc space over time, and failure to achieve/preserve segmental lordosis [17], [18], [19], [20], [21].

Expandable interbody devices can mitigate these risks by allowing a reduced initial cage size before expansion within the confines of the interbody space. This can lead to improved correction of disc height and foraminal volume, preservation of endplate integrity, decreased risk of neural injury, decreased risk of postoperative expulsion, and perhaps most importantly, maintenance or improvement in segmental lordosis and sagittal balance [22]. A variety of expandable cages have been designed but few allow lordotic expansion with a relatively large footprint [23,24]. This study evaluates the radiographic and clinical results of TLIF in patients using a novel articulating and expandable cage (FLXfit15). To our knowledge, FLXfit15 is the only expandable interbody that both articulates with a hinge mechanism to create a larger effective footprint and then expands in lordosis. This allows for more endplate contact area, potentially reducing subsidence, and offers up to 15 degrees of lordotic expansion.

The aim of this study was to document clinical and radiographic outcomes over 2 years postsurgery, with specific reference to pelvic parameters and postoperative subsidence. This manuscript is written following the STROBE checklist (Appendix A).

## Methods

### Patient population

**Fig. 1:**
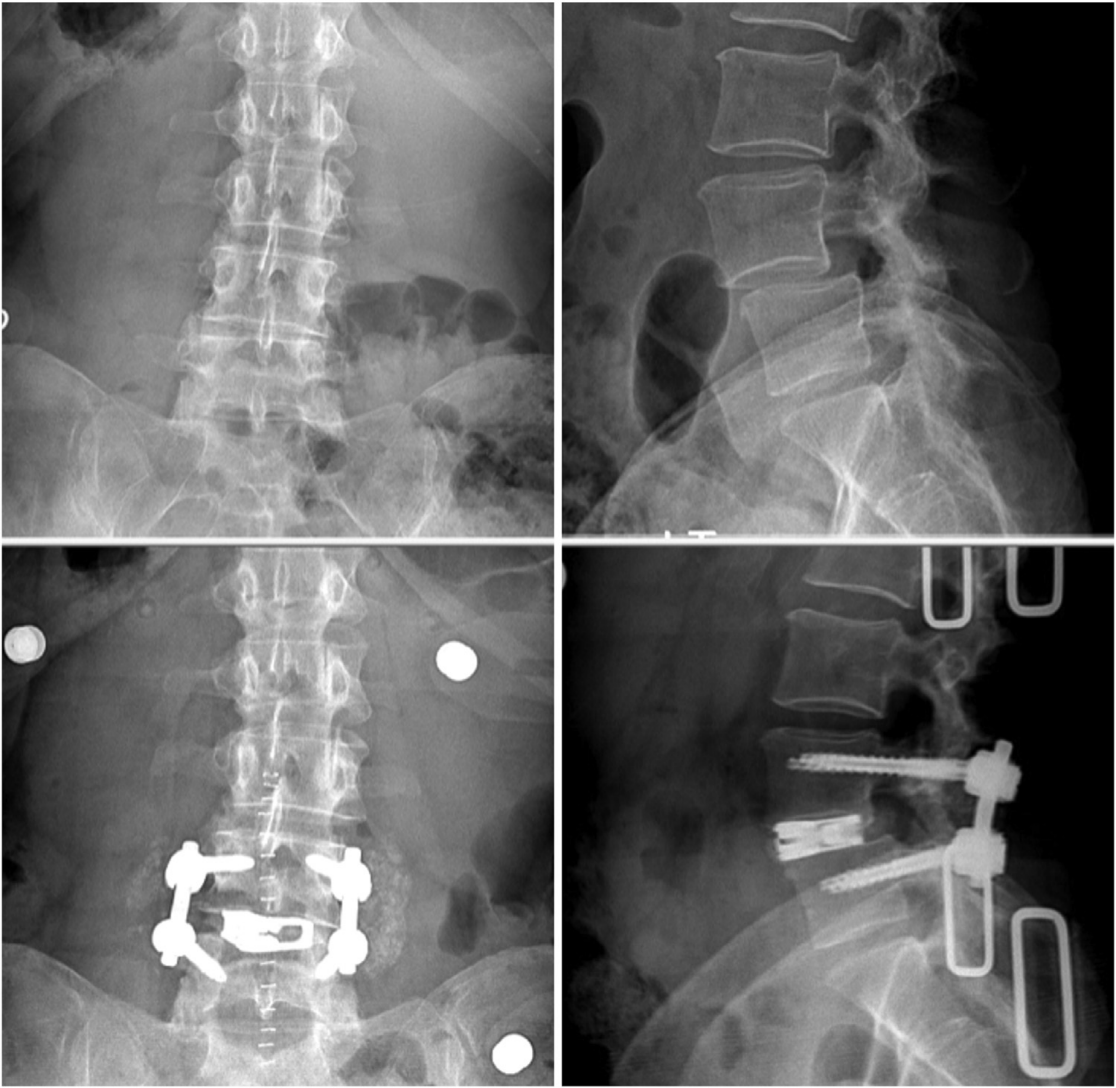
Pre- and postoperative pairs of radiographs showing the device placed at the L4/5 level.

This is an IRB-approved, prospective observational study performed on the cohort of consecutively enrolled patients who underwent single or 2 level TLIF procedure by a single surgeon, single institution using an expandible interbody cage (Fig. 1; FLXFit15, CoreLink Surgical, St. Louis, MO).

Inclusion criteria were age greater than 18 years, preoperative diagnosis of degenerative disc disease at one or two contiguous levels with or without symptomatic lumbar spondylolisthesis (up to grade 1) and failed conservative treatment (at minimum duration of 6 months). All patients had radiculopathy or neurogenic claudication with central spinal or neuroforaminal stenosis with or without spondylolisthesis. Patients had varying degrees of low back pain. Degenerative disc changes were observed on imaging and included loss of disc height, facet arthritis, modic endplate changes, and peridiscal osteophytes.

Exclusion criteria were BMI>40, trauma, inflammatory arthritis, pregnancy, prior fusion, and tumor. There were 4 people diagnosed with osteoporosis and one patient diagnosed with osteopenia in the study population (12.5% and 2.5%). Twenty patients never smoked, 15 patients were former smokers, and 5 patients were smokers (50%, 37.5%, and 12.5%)

### Surgical technique

All procedures were done as a standard open transforaminal lumbar interbody fusion. After induction of general anesthesia, the patient was placed prone on a well-padded 4-poster frame. The hips were extended creating a lordosing force on the lumbar spine. The patient was prepped and draped in typical sterile fashion and antibiotics were administered per hospital protocol. The dissection was carried subperiosteally to the tips of the transverse processes and sacral ala. The transverse processes, lateral facets, and sacral ala were decorticated with a high-speed burr. A laminectomy was performed, and neural decompression was completed. Next, a facetectomy was performed and the disc space was exposed on the most symptomatic side. The neural elements were retracted and an annulectomy was performed. Next, a discectomy was performed using pituitaries and disc space shavers. Significant attempt was made to remove as much disk as possible.

The end plates were then prepared with a rasp and angled osteotomes. Local bone graft from the laminectomy was mixed with allograft bone dust and approximately 3 mL were placed through a funnel into the anterior disc space. If necessary, a facetectomy was performed on the contralateral side. Distraction was placed across the disc space with a large lamina spreader with the teeth place on the adjacent lamina. If this was not possible due to anatomic constraints, a disc space distractor was temporarily placed on the contralateral side through an annulotomy. Next, the interbody cage was packed with the bone graft mixed as described above and then placed in the interbody space, across the mid-line due to the unique articulating design and rested on the apophyseal ring ventrally.

The cage was then fully expanded in lordosis. The bone graft mix was also packed dorsal to the cage. Pedicle screws were positioned in typical fashion. The remaining bone graft was combined with rhBMP-2 on an absorbable collagen sponge, rolled like a “burrito,” and then placed over the contralateral transverse processes and lateral facets. Appropriate size rods were selected and contoured, placed in the tulip heads, and then tightened in compression using the manufactures recommended torque. The wound bed was irrigated with saline and closed over a drain. Patients were admitted to the hospital and mobilized within 12 hours. Pre- and post-expansion cage height and any surgical complications were recorded.

### Clinical and radiographic measures

Demographic information for each patient, as well as clinical and radiographic evaluation, were collected at the pre-operative visit and at the two-week, 3-month, 6-month, 12-month, and 24-month postoperative visits. Patients were asked to complete the Oswestry disability index questionnaire (ODI) and the visual analogue pain score (VAS) for back and leg pain and undergo a standard set of radiographs (full-length standing AP and lateral spine radiographs).

**Fig. 2:**
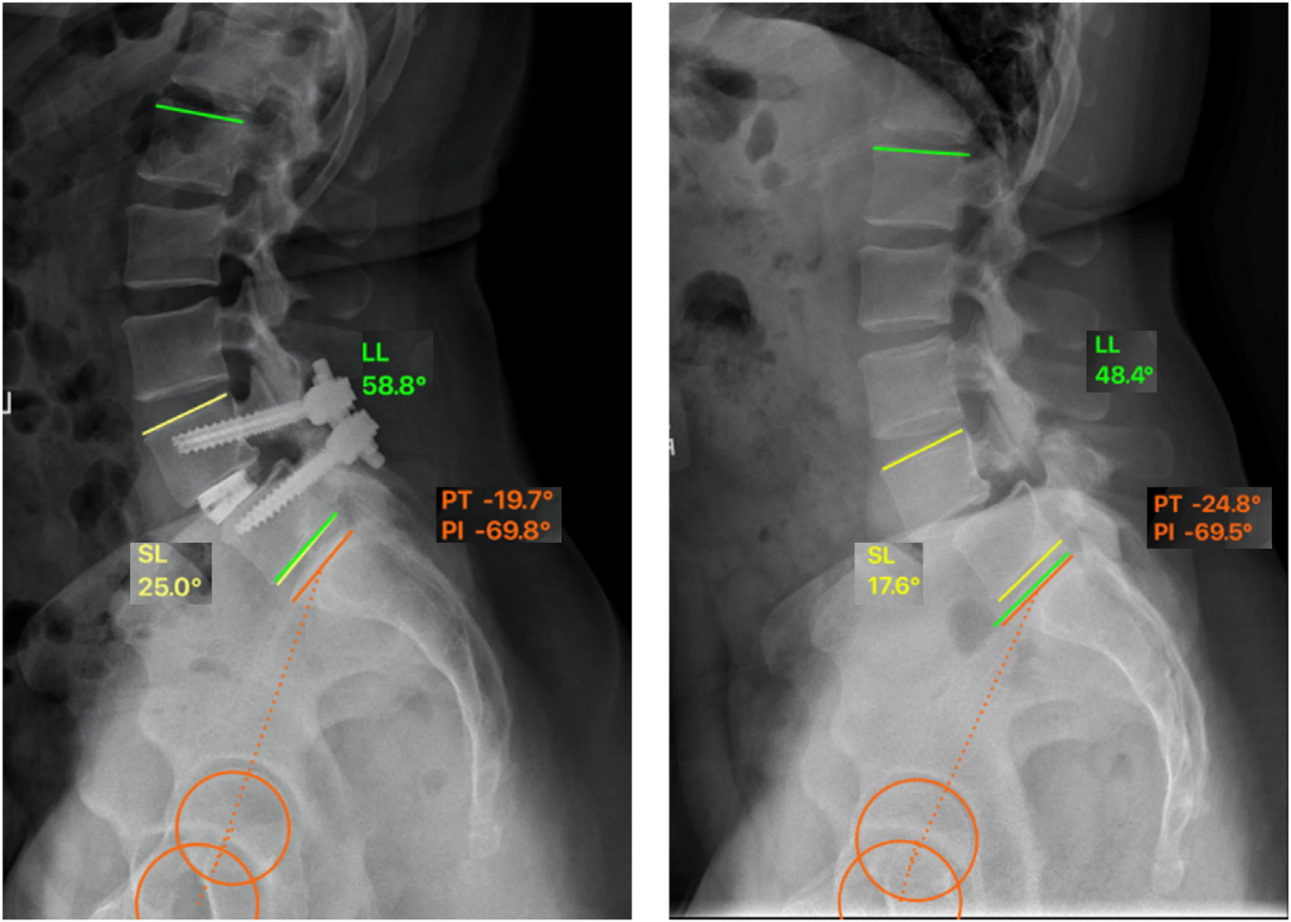
Evaluation of radiographic parameters (LL-lumbar lordosis, SL-segmental lordosis, PT-pelvic tilt, PI-pelvic incidence), DH-disc height was measured between each pair of evaluated operative levels as a distance at anterior and posterior side of the vertebrae.

Radiographic parameters including pelvic incidence (PI), pelvic tilt (PT), lumbar lordosis (L1-S-1, LL), segmental lordosis (SL), anterior, posterior, and average disc height (DH) at the operative level(s) were evaluated using Surgimap (Nemaris Inc., New York, NY) for every patient visit by a single independent operator (Fig. 2). Reliability of all radiographic measurements was verified by repeating a randomly selected set of measurements (20 cases for each measured parameter) by an additional observer and by the same observer after a washout period of at least two weeks. Subsidence of the cage was defined as any loss of disc space greater than 2 mm from 2 weeks to 24 months [25,26]. Lastly, fusion status was evaluated by the senior author at 12-month visit radiographically in combination with clinical symptoms that would suggest nonunion. All patients had posterolateral fusion as an adjunct and bridging posterolateral bone in the absence of motion on flexion/extension x-rays or hardware failure was considered pathognomonic for fusion. Similarly, bone growth in the interbody space connected the endplates in the absence of motion or hardware failure was considered fused.

### Statistical analysis

Means and standard deviations or medians and interquartile ranges were calculated for continuous variables and percentages for categorical variables. A paired *t* test or paired Wilcoxon signed rank test (where appropriate) were used to evaluate changes between the baseline (preoperative) values and individual post-operative values for each studied clinical and or radiographic variable. Minimal clinical important difference of 12.8 for ODI (100-point scale), 12 for VAS back pain (100-point scale) and 16 for VAS leg pain (100-point scale) were used for the population [27]. Inter/intra-rater evaluation of all radiographic parameters were assessed using interclass correlation coefficient. A-priori power determined that a minimum of 21 subjects (90% power) was needed to show the 25% improvement in disc height at the 6-month visit. Statistical significance was set at alpha=0.05.

## Results

### Demographics

**Fig. 3:**
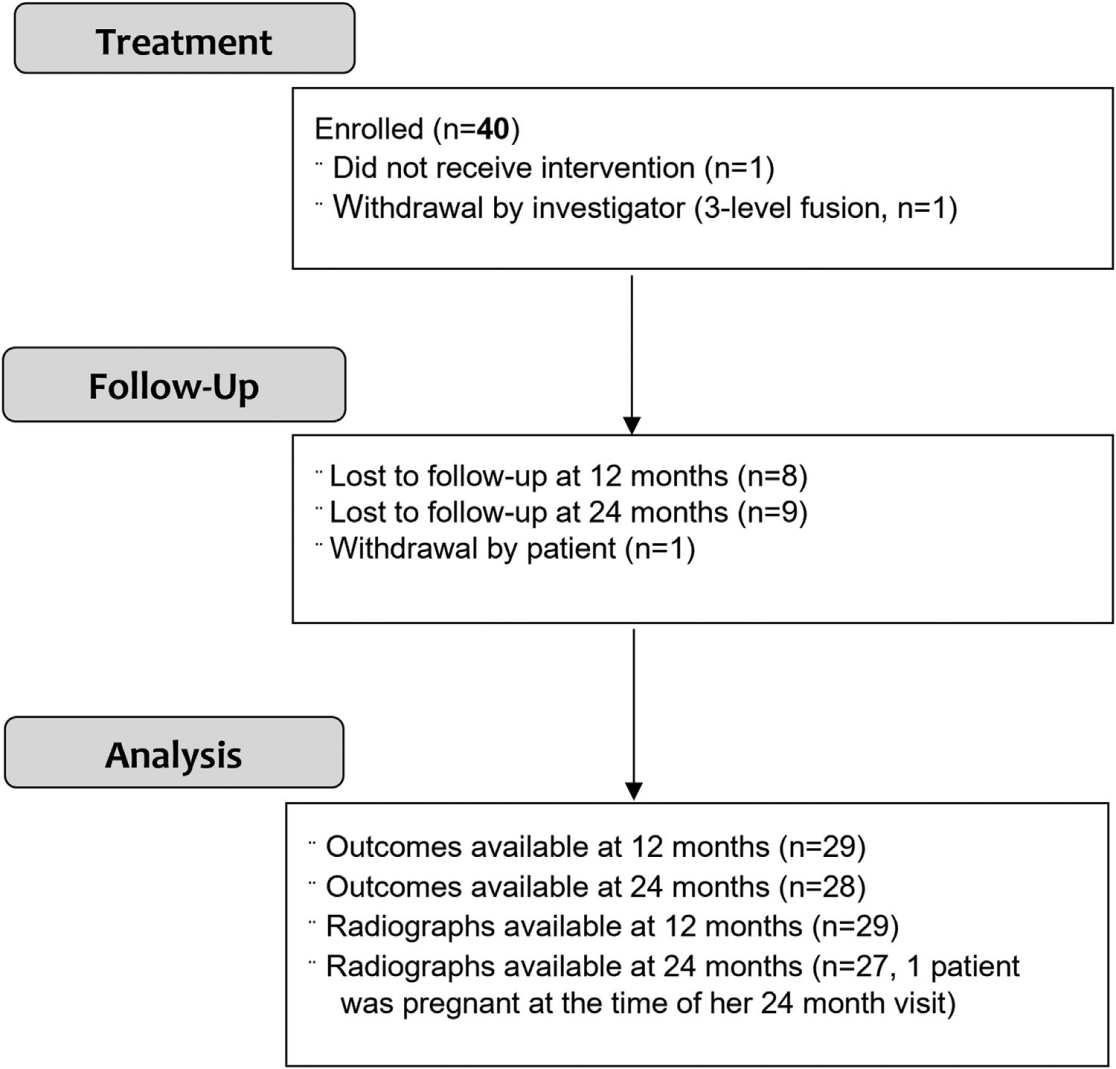
Study population – flow diagram.

A total of 40 patients were initially enrolled in the study. Two patients withdrew from the study, one before surgery and one after 6 months. One patient was excluded by the senior author due to fusion of more than 2 levels. A total of 37 patients underwent open single-level (n=23) or 2-level (n=14) TLIF with concomitant bilateral pedicle screws and posterolateral arthrodesis. In the single-level group, there were 5 cases performed at L3–L4, 11 cases at L4–L5, 7 cases at L5–S1. In the 2-level group, there were 14 cases performed at L4–L5 and L5–S1. The average age at the time of the surgery was 62 years (range, 32–79 years old), there were 19 females and 18 males with an average BMI of 30.8kg/m^2^ (range, 22.2–38.8). Average pre-expansion cage size was 9.4 mm (range, 8–13 mm) and then expanded to 12.8 mm on average (range, 10–16 mm). Four patients were lost to follow-up at 6 months, 8 patients were lost to follow-up at one year, 9 patients were lost to follow-up at 2 years (Fig. 3). A total of 28 patients were available for analysis of clinical and radiographic outcomes at 24-months (76% rate, see Appendix B for comparisons between study population and loss of follow up population).

### Clinical outcomes

**Fig. 4:**
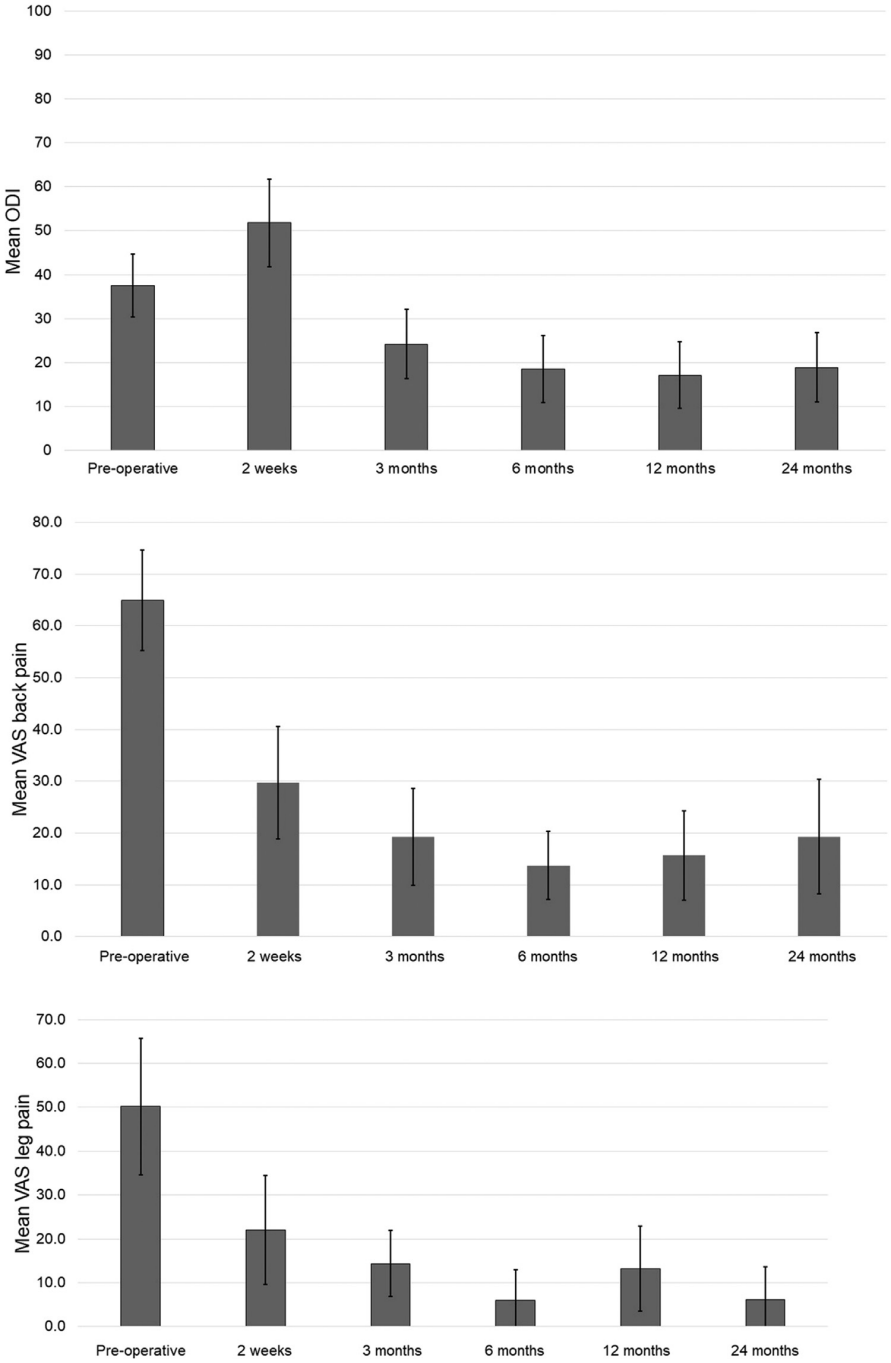
Clinical outcome measures. *Significant difference between baseline and postoperative value (<0.001).

Patients showed significant reduction in ODI, VAS back pain and VAS leg pain at 12-months and 24 months post-operatively (p<.001 for each, Table 1). Proportion of patients that achieved MCID for ODI, VAS back pain, and VAS leg pain was 66%, 83%, and 60% at one year, and 67%, 89%, and 70% at 2 years, respectively (Fig. 4).

**Table 1:** Average differences in clinical outcomes from baseline to 24 months.

	Baseline		at 12 months		p-value	Improvement	at 24 months		p-value	Improvement
	Mean	STD	Mean	STD			Mean	STD		
ODI	40.6	15.9	18.1	16.7	**<.001**	55%	19.8	19.4	**<.001**	51%
VAS back pain	65.5	22.6	16.8	20.4	**<.001**	74%	18.3	22.7	**<.001**	72%
VAS leg pain	51.7	32.4	15.4	24.0	**<.001**	70%	10.9	23.2	**<.001**	79%

ODI , Oswestry disability index; VAS, visual analogue pain score.

### Radiographic outcomes

**Fig. 5:**
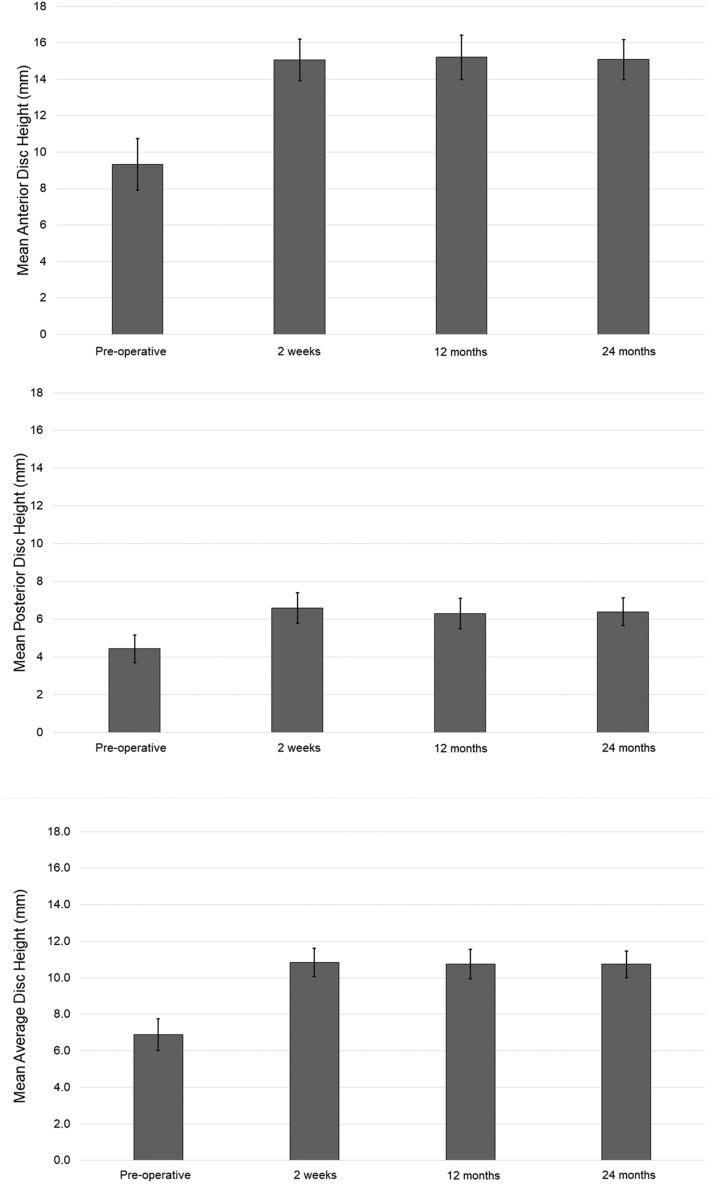
Radiographic outcome measures *Significant difference between baseline and postoperative value (<0.001).

Reliability of all radiographic measurements ranged from 0.694 to 0.879 for inter-operator and 0.654 to 0.811 for intraoperator. Both lumbar lordosis (LL: L1-S1) and segmental lordosis (SL) showed significant improvement at 12 months and 24 months (Table 2). LL improved on average by 13% at 1 year and 15% at two years, SL by 30% at one year and 23% at 2 years. Average DH increased by 53% at 1 year and 49% at 2 years (Fig. 5). There were no significant differences in PT and PI.

**Table 2:** Average differences in radiographic outcomes from baseline to 24 months.

	Baseline		at 12 mo		p-value	Delta	Improvement	at 24 mo		p-value	Delta	Improvement
	Mean	STD	Mean	STD				Mean	STD			
Segmental Lordosis (°)	21.2	11.1	27.6	9.3	**<.001**	6.4	30%	26.1	6.1	**.001**	5.3	23%
L1-S1 Lordosis (°)	47.1	19.6	53.1	14.1	**.020**	6.0	13%	54.0	11.7	**.049**	4.2	15%
Pelvic Incidence (°)	54.6	13.7	56.7	10.2	.317	2.2	4%	52.4	9.4	.721	0.8	-4%
Pelvic Slope (°)	22.4	10.2	21.0	7.2	.496	1.4	-6%	17.2	7.2	.168	3.1	-23%
Anterior Disc Height (mm)	9.6	3.9	15.3	3.4	**<.001**	2.4	59%	14.9	14.9	**<.001**	4.1	55%
Posterior Disc Height (mm)	4.7	1.9	6.4	2.1	**<.001**	3.4	37%	6.4	1.8	**<.001**	5.1	37%
Average Disc Height (mm)	7.1	2.4	10.9	2.2	**<.001**	4.4	53%	10.6	1.8	**<.001**	6.1	49%

### Complications

All patients achieved radiographically confirmed fusion at 12-months. One patient had a superficial site infection that resolved with oral antibiotics, and another had an incidental durotomy that was repaired intraoperatively without further incident. No device related complications occurred, and there was no subsidence seen at 24-months. No patient required additional surgery for non-union or device-related issues.

## Discussion

It has become widely accepted that appropriate spinal balance after fusion leads to better long-term outcomes in patients undergoing fusions for spinal deformity [7], [8], [9]. The importance of maintaining appropriate lumbar lordosis has also been shown to improve outcomes in sort segment degenerative fusions [28]. Failure to achieve segmental lordosis has been hypothesized to lead to acceleration of adjacent segment disease and poor outcomes [9]. There are many factors that can result in achieving appropriate segmental lordosis in degenerative fusions. These include pre-operative anatomy, patient bone quality, surgical technique, cage placement, and cage type [18,^24^,^15^,[29], [30], [31]. Preoperative segmental lordosis is a strong predictor of postoperative segmental lordosis [32,33]. In low pre-operative lordosis patients both static and expandable cages can be effective in increasing segmental lordosis. In patients with medium to high segmental lordosis before surgery, the risk for decreased lordosis is greater and expandable implants have been shown better at alleviated this problem [33].

In addition, expandable interbody devices are advantageous in that they allow a smaller initial footprint when navigating through an often-small neuroforaminal entry site reducing risk of nerve root injury [28,[34], [35], [36]. They also allow for less damage to the vertebra endplate during insertion due to the smaller initial size of the implant [23]. Damage to the endplates during placement of the cage can result in cage loosening, subsidence, retropulsion, and ultimately non-union [37]. Finally, many cages allow lordotic expansion which lengthens the anterior column and provides additional segmental lordosis [14]. Increase in disc space height is achieved as in procedures with static cages but with a smaller initial implant height [28,[34], [35], [36]. It has been shown that increased segmental lordosis is achieved in static cage fusions with more anterior placement of the device, and this is likely the case with expandable cages [29], [30], [31],38]. The ability of the articulated cage design used in this study to be steered to the anterior ring apophysis likely contributed to the significant increase in post-operative segmental lordosis.

Prior studies of TLIF procedures with static cages questioned whether ultimately the procedure “induced” kyphosis [29]. Indeed, many studies showed no improvement or very modest improvement in segmental lordosis following TLIF [32,39]. The more recent application of expandable cages has only added to this controversy with regard to improvement in segmental lordosis. Published studies revealed changes from −1.2 degrees loss to 7.8 degrees improvement [40], [41], [42], [43], [44], [45], [46], [47]. For example, while showing excellent clinical outcomes and disc height improvement in a study of MIS-TLIF with expandable oblique straight cages in patients with degenerative disc disease, there was no improvement noted in segmental lordosis [34].

Our study demonstrated an average increase in segmental lordosis in all patients of 5.3 degrees. This is a more favorable result when compared with other TLIFs with expandable designs (Table 3). Conclusions in determining the most efficacious devices for restoring or maintaining segmental lordosis as a result of TLIF are difficult given the marked heterogeneity of the literature. However, a recent meta-analysis [36] of local lordosis following TLIF comparing static to expandable cages favored expandable devices. Factors that complicate the study of this topic include differences in surgical technique (MIS vs. OPEN, unilateral vs. bilateral facetectomy), cage design (straight vs. banana-shaped), cage placement (anterior vs oblique vs. direct posterior), patient factors (age/bone quality, diagnosis), and surgical level fused.

**Table 3:** Comparison between previous studies and a current study.

Study	# Patients	Age (years)	Sex (M/F)	Length of FU		Clinical Outcomes				Radiographic Outcomes			
						Parameter	ODI	VAS P Leg	VAS P Back	Parameter	Lordosis	Disc Height	Subsidence
Current Study	37	62±10	18/19	Reported	24	MCID	67%	70%	89%	Preop	47.1±19.6 21.2±11.1§	7.1±2.4	0 (0%)
						Preop	40.6±15.9	51.7±32.4	65.5±22.6	Postop	54.0±11.7 26.1±6.1§	10.6±1.8	
				Average	25.2±3.1	Postop	19.8±19.4	10.9±23.2	18.3±22.7	Δ	4.2±10.8 5.3±6.6§	6.1±2.2	
						Δ	18.6±20.2	41.1±40.7	43.9±27.4				
(35)	49	60±16	21/28	Reported	-	MCID	64%	52%	52%	Preop	45.8±2.73^▲^	6.5±0.62^▲^	0 (0%)
						Preop	44±3.2	4.5±0.5^‡▲^	6.4±0.6^‡▲^	Postop	48.0±2.14^▲^	8.2±0.6^▲^	
				Average	19.3±6.4	Postop	24.2±3.8	1.9±0.5^‡▲^	3.1±0.6^‡▲^	Δ	-	-	
						Δ	-	-	-				
(25)	53	62±9	21/29	Reported	12	MCID	-	-	-	Preop	46.16±19.1	-	10 (20%)
						Preop	'-	-	-	Postop	49.61±17.3	-	
				Average	2.08±1.3^†^	Postop	25.96±19.9	17.9±26.9	25.5±27.8	Δ	3.81±15.6^§^	-	
						Δ	-	-	-				
(36)	129	61±13	57/72	Reported	-	MCID	58%	71%	76%	Preop	-	-	0 (0%)
						Preop	-	-	-	Postop	-	-	
				Average	4.4±3.8	Postop	-	-	-	Δ	-	-	
						Δ	14.6±19.1	3.9±3.4^‡^	3.4±2.6^‡^				
(26)	13	60±14	5/8	Reported	-	MCID	-	-	-	Preop	5.1±6.0^§^	5.7±1.5	0 (0%)
						Preop	-	5.1±3.0^‡^	7.0±2.9^‡^	Postop	6.8±4.7^§^	7.8±1.0	
				Average	7.5±27	Postop	-	1.1±1.7^‡^	3.1±2.9^‡^	Δ	1.7^§^	2.1	
						Δ	-	-	-				
(37)	54	56±13	24/30	Reported	24	MCID	-	-	-	Preop	40.9±15.7	8.3±3	0 (0%)
						Preop	61.4±17	7.8±2.5^‡^	7.7±2.3^‡^	Postop	45.4±16	13.3±2.6	
				Average	23.5	Postop	38.3±22.1	3.7±3.2^‡^	4.6±3.3^‡^	Δ	-	-	
						Δ	-	-	-				
(27)	35	61±12	17/18	Reported	24	MCID	-	-	-	Preop	8.1±6.7^§^	7.0±3.2	3 (7%)
						Preop	62.5±22	7.5±2.0^‡^	7.5±2.0^‡^	Postop	9.9±6.0^§^	11.3±2.2	
				Average	-	Postop	11.1±9.4	1.0±1.1^‡^	1.0±1.1^‡^	Δ	1.8±3.4^§^	4.3±3.6	
						Δ	51.4±25.1	6.5±2.5^‡^	6.5±2.5^‡^				
(28)	37	60±12	23/14	Reported	12	MCID	-	-	-	Preop	-	-	1 (2%)
						Preop	-	-	-	Postop	-	-	
				Average	-	Postop	-	-	-	Δ	3.7±2.9	4.2±3.8	
						Δ	63.2±13.2	6.7±2.6	6.7±2.6				
(29)	30	52±12	23/7	Reported	6	MCID	60%	70%	66.7%	Preop	52.4±10.5	9.5±2.1	-
						Preop	40.1±16.2	6.0±2.7^‡^	6.8±2.5^‡^	Postop	54.9±10.5	13.7±2.3	
				Average	-	Postop	20.4±16.8	2.9±2.8^‡^	3.7±3.0^‡^	Δ	-	4.2	
						Δ	-	-	-				
(30)	50	59	23/27	Reported	24	MCID	-	-	-	Preop	6.2^§^	5.6	1 (2%)
						Preop	59	81	78	Postop	14^§^	13.3	
				Average	-	Postop	23	16	26	Δ	-	-	
						Δ	36	65	52				
(38)	41	63±12	12/29	Reported	12	MCID	-	-	-	Preop	40.3±14.8	6.9±3.2	-
						Preop	-	-	-	Postop	42.7±13.9	8.3±2.4	
				Average	24.3±11.2	Postop	-	-	-	Δ	0.3±11.9	1.3±2.5	
						Δ	20.8	3.3	4.2				
(31)	40	54±13	11/29	Reported	24	MCID	-	-	-	Preop	-	7.3±2.6	0 (0%)
						Preop	-	33.4±33.5	57.9±27.6	Postop	-	12.5±1.2	
				Average	-	Post-op	-	9.0±1972	23.7±28.1	Δ	-	-	
						Δ	-	-	-				
(39)	32	59±16	20/12	Reported	3	MCID	72%	93%	93%	Preop	-	-	0 (0%)
						Preop	-	-	-	Postop	-	-	
				Average	-	Postop	-	-	-	Δ	1.6±3.7	4.5±2.5	
						Δ	-	-	-				
(32)	28	64±10	15/13	Reported	-	MCID	-	-	-	Preop	52.2±12.2	7.8±2.5	-
						Preop	32.2±7.97	-	-	Postop	56.9±11.4	16.0±3.2	
				Average	7.1±4.2	Postop	10.9±10.5	-	-	Δ	-	8.1±0.6	
						Δ	22.3±12.4	-	-				

*Note:* Length of Follow up is reported in months, except for instances marked with (^†^ - reported in years); all results are reported as mean and std. deviation, except for instances marked with (^▲^ - mean and std. error); VAS Pain for leg and back are reported in 100-point scale, except for instances marked with (^‡^ -10-point scale); Lordosis is reported as lumbar lordosis, except for instances marked with (^§^ - segmental lordosis); MCID – minimal clinically important difference

We have also shown improvement in disc height postsurgery which is maintained through 2-year follow-up. Both anterior and posterior disc height were significantly improved without evidence of subsidence. We hypothesize that the design features of the device contributed to this result. Both the articulated aspect that allows the cage to be steered anteriorly toward the apophyseal ring and the lordotic expansion are unique aspects that have been shown previously to be advantageous in preventing subsidence. The force of expansion is applied mostly to the anterior aspect of the apophyseal ring which may contribute to that lack of significant subsidence noted in this study. Other studies with a variety of devices have shown subsidence rates of 0% to 20% [40], [41], [42],[44], [45], [46], [47], [48], [49], [50], [51], [52].

Our excellent clinical results are comparable to the literature on the generally good results of interbody fusion for degenerative spine conditions (Table 3). Notably, there was significant improvement in leg pain indicating no significant neuropraxia or nerve damage as a result of cage placement through Kambin's triangle. Fusion rates were high with no patient experiencing pseudarthrosis or return to the operating room for hardware issues. The high fusion rate is possibly a result of the use of both interbody as well as bilateral posterolateral arthrodesis and the off-label use of rhBMP-2 in the posterolateral space. Cage design can affect lordosis in the fused degenerative segment [14]. Banana-shaped cages resulted in increased segmental lordosis in many studies [14]. This is likely a result of the more anterior placement of the device which is known to result in greater ability to achieve lordosis [38].

Our average increase in segmental lordosis is notably greater than studies with linear expandable cages. Yee et al. [39] showed no difference in segmental lordosis after use of expandable cages versus static cages. Indeed, they noted improvement of only 1 to 3 degrees on average. Unfortunately, this study examined multiple expandable interbody devices including nonlordotic expanding cages as well as linear and banana-shaped implants. Certain cage designs and placement may also affect subsidence [14]. Stickely et al. [53] demonstrated increased risk of subsidence in a study comparing expandable devices to the use of static cages. Confounding their results, however, was the use of multiple expandable devices, greater inclusion of MIS-TLIF in the expandable group (88 vs. 26%) and less use of rhBMP-2 in the static group (32 vs. 60%).

Our operative technique was to perform more traditional open TLIF procedures. This allowed for bilateral facetectomy when required and perhaps contributed to the significant improvement in segmental lordosis. Removing both facet joints with discectomy improves the ability to obtain segmental lordosis by mobilizing the functional spinal unit [30]. The addition of an anterior column lengthening with a cage and subsequent posterior compression of the pedicle screws creates a favorable lordotic force for correction of segmental lordosis [15,^30^,^54^,55]. In MIS-TLIF, bilateral facetectomy is not typically performed and the ability to mobilize the segment and compress the screws is reduced [34]. In fact, some studies have shown less ability of MIS-TLIF to improve or maintain segmental lordosis and potential for increased subsidence in more rigid spines [32,34].

The literature suggests that a unilateral minimally invasive, obliquely oriented, and posteriorly placed static cage is not the best option to achieve lordosis in the fused segment. In the end, it is likely that maintaining or improving segmental lordosis as an outcome of a TLIF procedure is multifactorial [30]. The expandable interbody cage is one tool to aid in this endeavor. Preoperative segmental lordosis is related to postoperative segmental lordosis achieved and inversely related to the amount of the increase in lordosis [32,33]. Our results reflect this finding as well.

### Strengths and limitations

The strengths of this study are that it is a single surgeon consecutive patient series, the surgical procedures were consistent, all TLIFs performed during enrollment used this specific interbody cage, and the patients were followed prospectively to at least 2 years so radiographic findings are conclusive. Some of the limitations arise from the fairly heterogenous patient diagnoses and anatomic location of the surgical procedure. Other fusion procedures were performed during the enrollment period so selection bias for TLIF was introduced. The number of patients included was also relatively small but statistical significance was still valid for most measures. Lastly, this study reports on the loss of follow-up with a retention of 76% of the study cohort. Appendix B compares selected variables between the study cohort and a loss of follow up cohort with no statistical significance noted between the two groups.

## Conclusion

Preoperative planning is paramount to fusions for degenerative spine disease. When restoring segmental lordosis is important, an articulated lordotic expanding interbody device is a useful adjunct to surgical technique. The importance of maintaining and, when necessary, improving the segmental lordosis during degenerative fusions is borne out in the long term as potential for reduction or delay in adjacent segment disease. The expandable device used in this study with the operative techniques as described enables increased segmental lordosis with good clinical outcomes and no significant subsidence up to 2 years postsurgery. Further studies are required to determine the most appropriate device for more specific clinical scenarios and the effect on the durability of the surgical results.

## Declaration of Competing Interest

One or more authors declare potential competing financial interests or personal relationships as specified on required ICMJE-NASSJ Disclosure Forms.

## Appendix Supplementary materials

Appendix A: STROBE checklist Image, application 1

Appendix B: Comparison of baseline demographics between the study group and loss to follow-up group Image, application 2
