# Supplementary material for: Transforaminal lumbar interbody fusion with an expandable interbody device: Two-year clinical and radiographic outcomes
Source: N Am Spine Soc J. 2023 Oct 13;16:100286. doi: 10.1016/j.xnsj.2023.100286 (PMC10652136; doi:10.1016/j.xnsj.2023.100286)
Supplement: Supplementary file 2 — Appendix B: Comparison of baseline demographics between the study group and loss to follow-up group [file mmc2.docx]

Appendix B

|  | at 12 months | | | | | at 24 months | | | | | Total | |
| --- | --- | --- | --- | --- | --- | --- | --- | --- | --- | --- | --- | --- |
|  | Study Group (n=29) | | LOF Group (n=8) | | p-value | Study Group (n=28) | | LOF Group (n=9) | | p-value |  |  |
|  | Mean | STD | Mean | STD |  | Mean | STD | Mean | STD |  | Mean | STD |
| Age (years) | 62.0 | 10.0 | 61.0 | 9.0 | 0.734 | 63.3 | 9.5 | 56.9 | 10.3 | 0.095 | 61.7 | 9.9 |
| Height (inch) | 66.9 | 4.1 | 67.1 | 3.9 | 0.631 | 67.4 | 4.4 | 66.1 | 2.5 | 0.423 | 67.1 | 4.0 |
| Weight (lb) | 202.0 | 45.0 | 191.0 | 32.0 | 0.522 | 198.9 | 43.3 | 201.4 | 42.1 | 0.876 | 199.5 | 42.4 |
| BMI (kg/m^2^) | 31.2 | 5.6 | 29.3 | 4.3 | 0.366 | 30.7 | 5.7 | 31.0 | 4.3 | 0.882 | 30.8 | 5.3 |

| Variable | | at 12 months | | | | | at 24 months | | | | | Total | |
| --- | --- | --- | --- | --- | --- | --- | --- | --- | --- | --- | --- | --- | --- |
|  |  | Study Group (n=29) | | LOF Group (n=8) | | p-value | Study Group (n=28) | | LOF Group (n=9) | | p-value |  |  |
|  |  | Count | % | Count | % |  | Count | % | Count | % |  | Count | % |
| Levels | L3-L4 | 4 | 14% | 1 | 13% | 1 | 3 | 11% | 2 | 22% | 0.3 | 5 | 14% |
|  | L4-L5 | 9 | 31% | 2 | 25% |  | 10 | 36% | 1 | 11% |  | 11 | 30% |
|  | L4-L5, L5-S1 | 11 | 38% | 3 | 38% |  | 11 | 39% | 3 | 33% |  | 14 | 38% |
|  | L5-S1 | 5 | 17% | 2 | 25% |  | 4 | 14% | 3 | 33% |  | 7 | 19% |
| SEX | F | 15 | 52% | 4 | 50% | 0.9 | 13 | 46% | 6 | 67% | 0.3 | 19 | 51% |
|  | M | 14 | 48% | 4 | 50% |  | 15 | 54% | 3 | 33% |  | 18 | 49% |
